# T-cell engagers: model interrogation as a tool to quantify the interplay of relative affinity and target expression on trimer formation

**DOI:** 10.3389/fphar.2024.1470595

**Published:** 2024-10-08

**Authors:** Massimo Lai, Cesar Pichardo-Almarza, Meghna Verma, Md Shahinuzzaman, Xu Zhu, Holly Kimko

**Affiliations:** ^1^ Clinical Pharmacology and Quantitative Pharmacology, R&D Biopharmaceuticals, AstraZeneca plc, Cambridge, United Kingdom; ^2^ Clinical Pharmacology and Quantitative Pharmacology, R&D Biopharmaceuticals, AstraZeneca Plc, Gaithersburg, MD, United States; ^3^ Clinical Pharmacology and Quantitative Pharmacology, R&D Biopharmaceuticals, AstraZeneca Plc, Waltham, MA, United States

**Keywords:** T-cell engagers, TCEs, TCR, TAA, dose-response, target engagement, trimer formation, bispecifics

## Abstract

T-cell engagers (TCEs) represent a promising therapeutic strategy for various cancers and autoimmune disorders. These bispecific antibodies act as bridges, connecting T-cell receptors (TCRs) to target cells (either malignant or autoreactive) via interactions with specific tumour-associated antigens (TAAs) or autoantigens to form trimeric synapses, or trimers, that co-localise T-cells with target cells and stimulate their cytotoxic function. Bispecific TCEs are expected to exhibit a bell-shaped dose-response curve, with a defined optimal TCE exposure for maximizing trimer formation. The shape of the dose-response is determined by a non-trivial interplay of binding affinities, exposure and antigens expression levels. Furthermore, excessively low binding to the TCR may reduce efficacy, but mitigate risk of over-stimulating cytokine secretion or induce effector cell exhaustion. These inevitable trade-off highlights the importance of quantitatively understanding the relationship between TCE concentration, target expression, binding affinities, and trimer formation. We utilized a mechanistic target engagement model to show that, if the TCE design parameters are close to the recommended ranges found in the literature, relative affinities for TCR, TAA and target expression levels have qualitatively different, but predictable, effects on the resulting dose-response curve: higher expression levels shift the curve upwards, higher antigen affinity shifts the curve to the left, and higher TCR affinity shifts the curve upwards and to the left.

## 1 Introduction

Bispecific immune cell therapies were originally proposed by (Nisonoff and Rivers, 1961) falling within the class of immuno-oncology therapies wherein one binding domain that targets the cancer antigen and the other targets the T cell receptor (TCR). The involvement of the *TCR* complex facilitates the recognition, and mediates the redirected lysis, of the cancer cells by T cells. The perforin and granzymes released by the effector cells downstream of the activation of the T cells, results into the apoptosis and killing of the targeted cancer cells. Typically, TCEs are designed with low-affinity binding to the TCR, in the micromolar range, with higher affinity for the tumour-associated antigens (TAAs), in the nanomolar range. The formation of “trimers” (that is to say, a TCR-TCE-TAA trimeric synapse that cross-bridges the effector cell and the tumor cell) is widely understood to be a key driver of cytotoxic function and therefore efficacy, by forming artificial immunological synapses (Betts et al., 2019; Flowers et al., 2023). Bispecific molecules that can form ternary complexes (or trimers) are known to exhibit a bell-shaped dose-response, something that was both predicted by mathematical arguments and confirmed experimentally Douglass et al. (2013); Lin and Chen (2021); Granger et al. (2021). At this point in time, measurement of trimeric synapses *in vivo* is still challenging, and can only be inferred indirectly from *in-vitro* assays. Direct absolute quantification of this important biomarker in a clinical setting is still elusive, and that leaves mechanistic modeling as an avenue to help predict clinically efficacious doses, and minimize costly and wasteful trial-and-error at the clinical trial stage. We may speculate that *in vitro* trimer quantification could be obtained by fluorescence imaging methods similar to those employed in the study of other compounds whose mechanism of action relies on ternary complexes, such as PROTACs. A potential avenue could be the use of something akin to AlphaLISA or TR-FRET fluorescence essays, where the simultaneous signal of activated bound fluorophores brought into close proximity is detected, as reviewed for example in Casement et al. (2021). Alternatively, other authors have used cross-linking assays that relied on flow cytometry Qi et al. (2018). While sample collection in a clinical setting may be impossible, translatable insight could be gained by exploiting the recent progresses in the rapidly developing field of micro-physiological systems (MPS), where human-derived tissue is cultured *in vitro* Virumbrales-Muñoz and Ayuso (2022).

While the impact of relative affinity of TCE design has been pointed out by other authors (Vafa and Trinklein, 2020), due to the complex interactions involved in TCE with different cell types, number of receptors, binding affinity, a quantitative understanding of the interplay between these parameters is challenging. A higher affinity for the TCR would have a theoretically positive impact on trimer formation, but at the expense of increased risk of cytokine release, increased risk of T-cell exhaustion (Zhang et al., 2024) and biodistribution into lymphocyte-rich tissues at the expenses of the tumor tissue, thereby reducing local exposure (Vafa and Trinklein, 2020). However, erring on the side of caution with respect to cytokine release may lead us to select a TCR affinity too low for efficacy. Conversely, excessively high affinity for the TAA may improve trimer formation, but also increase internalisation by tumor cells and reduce local exposure by decreasing the TCE’s half-life (Mandikian et al., 2018). In the face of such non-trivial trade-offs, quantitative mathematical modeling can be helpful for guiding a rational selection of compound parameters that determine efficacy and safety.

This study is a first attempt to explore the relationship between TCE concentration and trimer formation under varying target cancer receptor expression levels. We advocate both continued development of more accurate models, and model interrogation to assist design choices. For the sake of simplicity, we are focusing on target engagement, in particular on the impact of relative affinity and local exposure on the formation of trimers, which are the fundamental driver of cytotoxic function. As a test-bench, we selected the case of a bispecific TCE targeting TAAs at baseline B-cells levels human lymph nodes, for which most relevant parameters (such as cell counts and surface receptors copy numbers) could be sourced from the literature (Ginaldi et al., 1998; Nerreter et al., 2019; Mandikian et al., 2018; Stulnig et al., 1995; Liu et al., 2000). The baseline values for TCR and TAA affinities where selected in accordance to the values recommended by Vafa and Trinklein (2020).

Models addressing specific indications, with different targets and/or different levels of disease burden, will have to rely on disease-specific biological and histological data to quantify cell counts and target expression levels.

## 2 Methods

### 2.1 Model formulation

**FIGURE 1:**
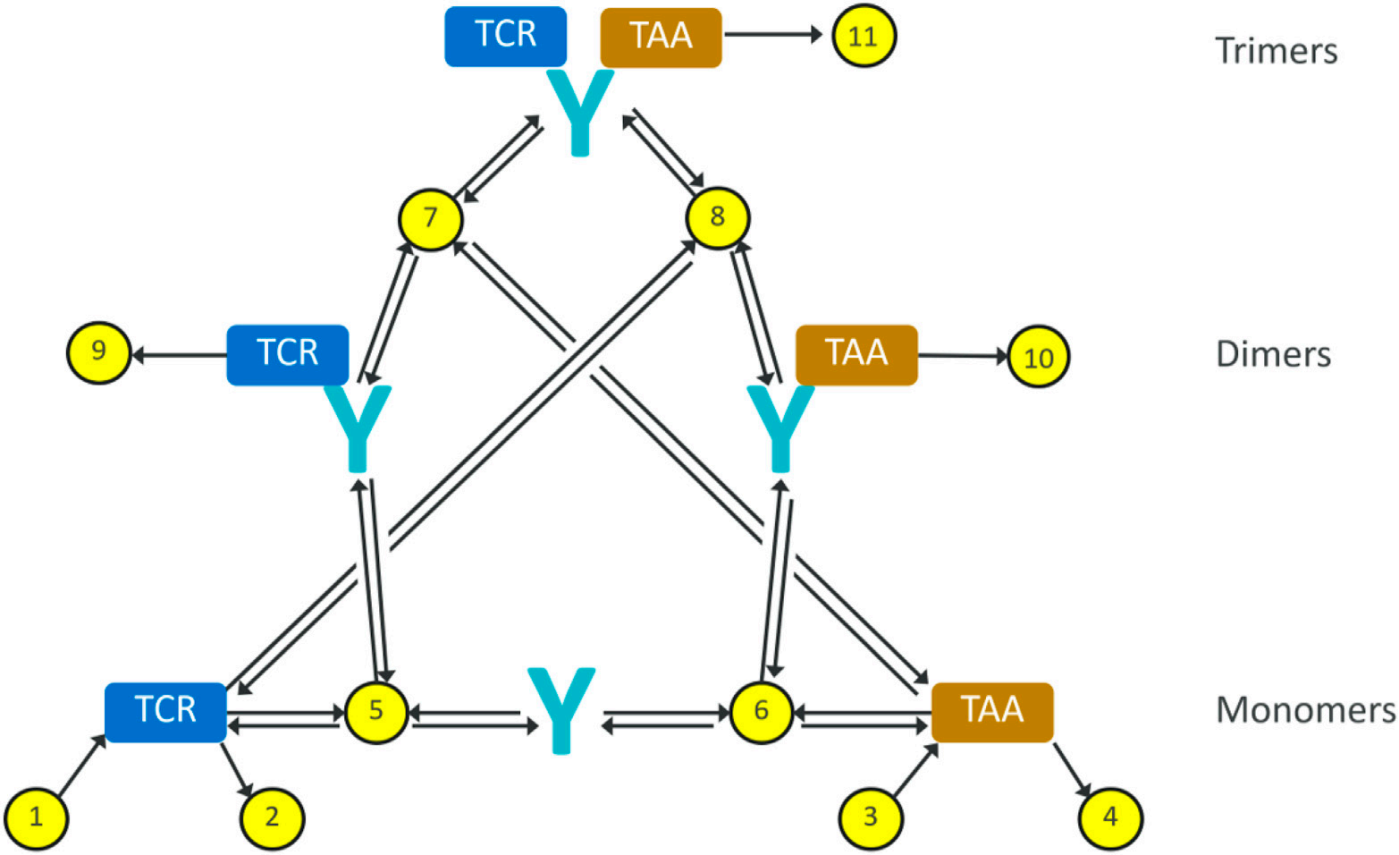
Reaction diagram for our minimal trimer formation/target engagement model. The yellow dots represent reactions, the black arrows represent reaction fluxes. Reactions 1–4 represent receptor turnover; reactions 5–8 represent non-covalent binding and dissociation; reactions 9–11 represent internalisation and degradation of receptor-bound antibodies.

In order to describe the interaction of a given TCE with the effector and cancer cells, a mathematical model is proposed based on a mass balance, and assuming a well-mixed compartment as simplifying assumption, where total levels of targets and antibody are considered. The resulting reaction diagram, describing all these interactions including the formation of dimers and trimers, is illustrated in Figure 1. The corresponding synthesis/degradation (Reactions 1–4), binding and internalization Reactions 5–8 and complex degradation Reactions 9–11 are the following::

∅→TCR (1)

TCR→∅ (2)

∅→TAA (3)

TAA→∅ (4)

TCE+TCR⇌TCE_TCR (5)

TCE+TAA⇌TCE_TAA (6)

TCE_TCR+TAA⇌TCE_TCR_TAA (7)

TCE_TAA+TCR⇌TCE_TCR_TAA (8)

TCE_TCR→∅ (9)

TCE_TAA→∅ (10)

TCE_TCR_TAA→∅ (11)

The model considers several components: monomers of TCE (Equation 12), TCR receptors on T-cells (Equation 13), TAA receptors on B-cells (Equation 14), dimers of TCE-TCR (Equation 15) and TCE-TAA (Equation 16), and the trimeric complex where all three bind together TCE-TCR-TAA (Equation 17). The corresponding system of ordinary differential equations is:

$$\begin{array}{cc} \frac{d\left[TCE\right]}{dt}= & -k_{on}^{\mathrm{TCR}}\cdot \left[TCE\right]\cdot \left[TCR\right]-k_{on}^{\mathrm{TAA}}\cdot \left[TCE\right]\cdot \left[TAA\right]\\ & +k_{\mathrm{off}}^{\mathrm{TCR}}\cdot \left[TCE\_TCR\right]+k_{\mathrm{off}}^{\mathrm{TAA}}\cdot \left[TCE\_TAA\right] \end{array}$$ (12)

$$\begin{array}{cc} \frac{d\left[TCR\right]}{dt}= & -k_{on}^{\mathrm{TCR}}\cdot \left[TCE\right]\cdot \left[TCR\right]-k_{on}^{\mathrm{TCR}}\cdot \left[TCE\_TAA\right]\cdot \left[TCR\right]\\ & +k_{\mathrm{off}}^{\mathrm{TCR}}\cdot \left[TCE\_TCR\right]+k_{\mathrm{off}}^{\mathrm{TCR}}\cdot \left[TCE\_TCR\_TAA\right]\\ & +k_{\mathrm{syn}}^{\mathrm{TCR}}-k_{\deg}^{\mathrm{TCR}}\cdot \left[TCR\right] \end{array}$$ (13)

$$\begin{array}{cc} \frac{d\left[TAA\right]}{dt}= & -k_{on}^{\mathrm{TAA}}\cdot \left[TCE\right]\cdot \left[TAA\right]-k_{on}^{\mathrm{TAA}}\cdot \left[TCE\_TCR\right]\cdot \left[TAA\right]\\ & +k_{\mathrm{off}}^{\mathrm{TAA}}\cdot \left[TCE\_TAA\right]+k_{\mathrm{off}}^{\mathrm{TAA}}\cdot \left[TCE\_TCR\_TAA\right]\\ & +k_{\mathrm{syn}}^{\mathrm{TAA}}-k_{\deg}^{\mathrm{TAA}}\cdot \left[TAA\right] \end{array}$$ (14)

$$\begin{array}{cc} \frac{d\left[TCE\_TCR\right]}{dt}= & +k_{on}^{\mathrm{TCR}}\cdot \left[TCE\right]\cdot \left[TCR\right]-k_{\mathrm{off}}^{\mathrm{TCR}}\cdot \left[TCE\_TCR\right]\\ & -k_{on}^{\mathrm{TAA}}\cdot \left[TCE\_TCR\right]\cdot \left[TAA\right]\\ & +k_{\mathrm{off}}^{\mathrm{TAA}}\cdot \left[TCE\_TCR\_TAA\right]-k_{\mathrm{int}}^{\mathrm{TCR}}\cdot \left[TCE\_TCR\right] \end{array}$$ (15)

$$\begin{array}{cc} \frac{d\left[TCE\_TAA\right]}{dt}= & +k_{on}^{\mathrm{TAA}}\cdot \left[TCE\right]\cdot \left[TAA\right]-k_{\mathrm{off}}^{\mathrm{TAA}}\cdot \left[TCE\_TAA\right]\\ & -k_{on}^{\mathrm{TCR}}\cdot \left[TCE\_TAA\right]\cdot \left[TCR\right]\\ & +k_{\mathrm{off}}^{\mathrm{TCR}}\cdot \left[TCE\_TCR\_TAA\right]-k_{\mathrm{int}}^{\mathrm{TAA}}\cdot \left[TCE\_TAA\right] \end{array}$$ (16)

$$\begin{array}{cc} \frac{d\left[TCE\_TCR\_TAA\right]}{dt}= & +k_{on}^{\mathrm{TCR}}\cdot \left[TCE\_TAA\right]\cdot \left[TCR\right]\\ & +k_{on}^{\mathrm{TAA}}\cdot \left[TCE\_TCR\right]\cdot \left[TAA\right]\\ & -k_{\mathrm{off}}^{\mathrm{TCR}}\cdot \left[TCE\_TCR\_TAA\right]\\ & -k_{\mathrm{off}}^{\mathrm{TAA}}\cdot \left[TCE\_TCR\_TAA\right]\\ & -k_{\mathrm{int}}^{\mathrm{Trm}}\cdot \left[TCE\_TCR\_TAA\right] \end{array}$$ (17)

As shown by García-Sánchez et al. (2023), in the case of the slow association rates $\left(k_{on}\leq 10^{6}M^{-1}s^{-1}\right)$ , 3D binding rate constants are a good approximations of 2D binding rate constants. Most antibodies seem to have $k_{on}$ that lie in the $10^{4}-10^{6}M^{-1}s^{-1}$ range, as discussed by (Liu et al., 2015; Schlosshauer and Baker, 2004).

### 2.2 Model implementation

The final model parameters are summarised in Table 1, including units, description and sources. The model was implemented in Python 3.9.7 and the ODEs were integrated using Numpy 1.26.4. All simulation plots were generated using Matplotlib 3.7.3.

**TABLE 1:** Summary of physiological parameters pertinent to the estimation of target abundance, and TCE binding parameters for the trimer formation model. In the absence of kinetic data, a plausible value of $10^{-3}\;nM^{-1}s^{-1}$ was used for the antibody-receptor association rate constant, chosen as the mid-point of the reported range for protein-protein interactions.

Parameter	Value	Units	Description	Source
Conc. lymphocytes	2.4e9	Cells/L	Lymphocytes in blood	Stulnig et al. (1995)
T cell fraction	0.85	Dim.less	Range (0.8–0.9)	Stulnig et al. (1995)
B cell fraction	0.15	Dim.less	Range (0.1–0.2)	Stulnig et al. (1995)
TCR per cell	$10^{5}$	copies/cell	TCR receptors per T cell	Liu et al. (2000)
TAA per cell	$10^{4}$	copies/cell	TAA copies per B cell	Ginaldi et al. (1998)
$K_{d}^{\mathrm{TCR}}$	$10^{3}$	nM	TCE affinity for TCR	Vafa and Trinklein (2020)
$K_{d}^{\mathrm{TAA}}$	1	nM	TCE affinity for TAA	Dreier et al. (2002)
$k_{on}^{\mathrm{TCR}}$	1e5	$M^{-1}s^{-1}$	Assoc. rate const. to TCR	Assumption
$k_{\mathrm{off}}^{\mathrm{TCR}}$	0.26	$s^{-1}$	Dissoc. rate const. to TCR	Calculated
$k_{on}^{\mathrm{TAA}}$	1e5	$M^{-1}s^{-1}$	Assoc. rate const. to TAA	Assumption
$k_{\mathrm{off}}^{\mathrm{TAA}}$	0.00149	$s^{-1}$	Dissoc. rate const. to TAA	Calculated
$k_{\mathrm{syn}}^{\mathrm{TCR}}$	1.329e-4	$nM\cdot s^{-1}$	Synthesis rate of TCR	Calculated
$k_{\deg}^{\mathrm{TCR}}$	1.834e-5	$s^{-1}$	Degradation rate of TCR	Hentati et al. (2010)
$k_{\mathrm{syn}}^{\mathrm{TAA}}$	7.691e-7	$nM\cdot s^{-1}$	Synthesis rate of TAA	Calculated
$k_{\deg}^{\mathrm{TAA}}$	1.834e-5	$s^{-1}$	Degradation rate of TAA	Assum. = TCR
$k_{\mathrm{int}}^{\mathrm{TCR}}$	5.501e-5	$s^{-1}$	Internal. rate of bound TCR	Liu et al. (2000)
$k_{\mathrm{int}}^{\mathrm{TAA}}$	5.501e-5	$s^{-1}$	Internal. rate of bound TAA	Assum. = TCR
$k_{\mathrm{int}}^{\mathrm{Trm}}$	1.834e-5	$s^{-1}$	Degr. rate of trimer	Assum. = TCR

We tested the plausibility of our steady-state approach by simulating time to steady state under different assumption with regard to the kinetics of target engagement and the concentrations of antibody. Under a wide range of conditions steady state was reached in 1–2 h. The only scenario where our approach may be unreliable is for exceptionally slow kinetics $\left(k_{on}<10^{4}M^{-1}s^{-1}\right)$ and sub-nanomolar exposure, where equilibration may take several hours (Supplementary Figure S1). Newly or recently developed TCEs typically have half-lives of about 1 week, clinical doses in the mg range, and have post-infusion Cmax in the 1–100 nM range (Hutchings et al., 2021; Ahn et al., 2023), conditions that would be within the boundaries of applicability of our approach. In particular, over a time of 24 h post infusion, the concentration of a TCE with a half-life of 7 days would fall by only about 10% (Supplementary Figure S2).

We also tested the robustness of the model with respect to parameter variability by performing 1,000 simulations where each parameter was allowed to randomly vary up to 2-fold from the baseline value. Only the independent parameters, i.e. parameters that were not known analytical functions of other parameters, were included in the perturbation study. The simulated curves can be found in the Supplementary Material (Supplementary Figure S3), and show that the qualitative shape of the steady-state dose-response curve remains the same.

## 3 Results

**FIGURE 2:**
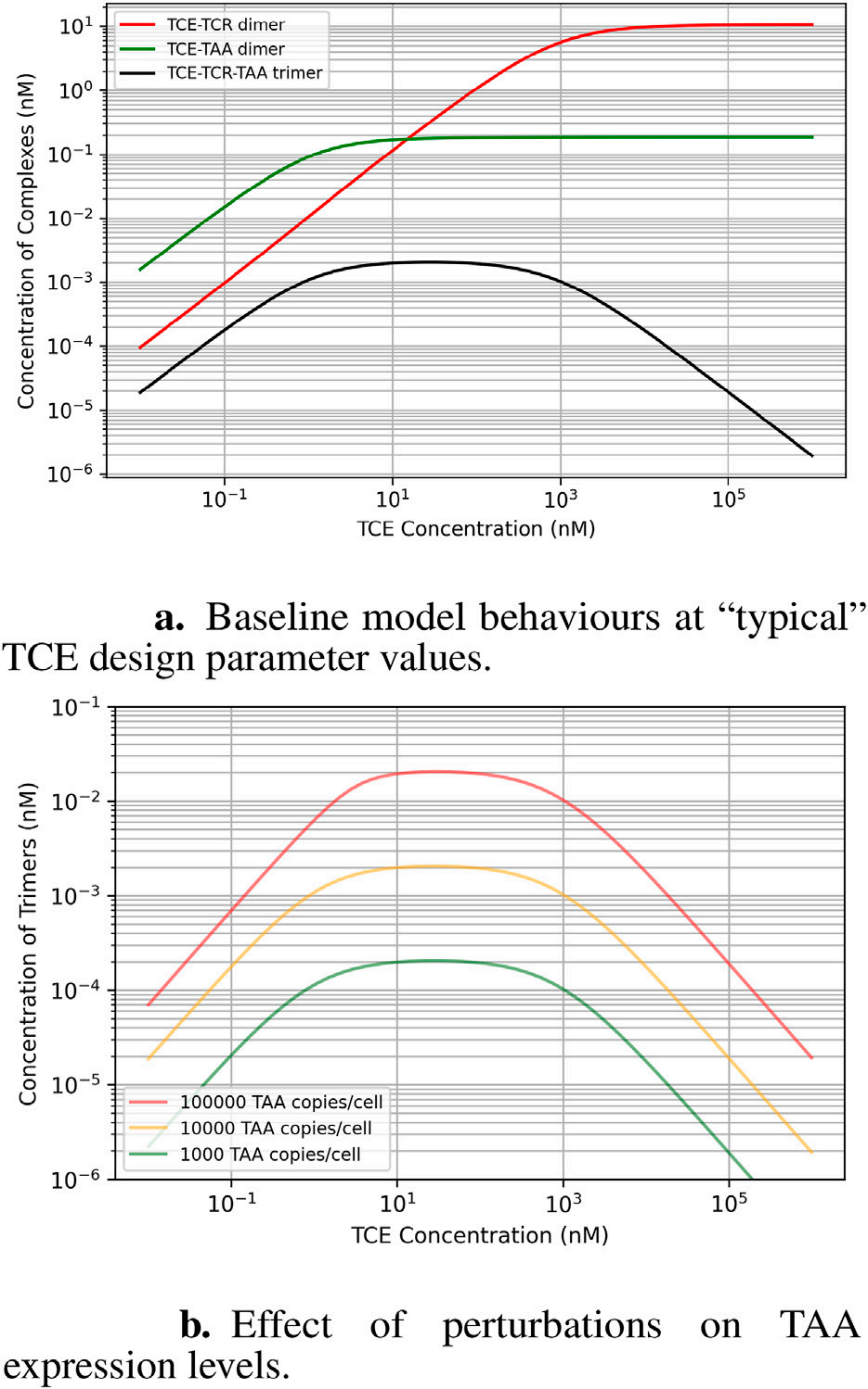
Baseline model behavior at the design parameter values recommended for TCEs (micromolar affinity for the TCR, robustly expressed at $10^{5}$ copies/cell, and nanomolar affinity for a TAA expressed at about $10^{4}$ copies/cell): **(A)** Steady-state dose-response of dimers and trimer concentrations at varying concentrations of antibody; **(B)** effect of variable TAA expression: the dose-response curve shifts vertically, but the exposure at which the efficacy plateau is reached remains the same.

**FIGURE 3:**
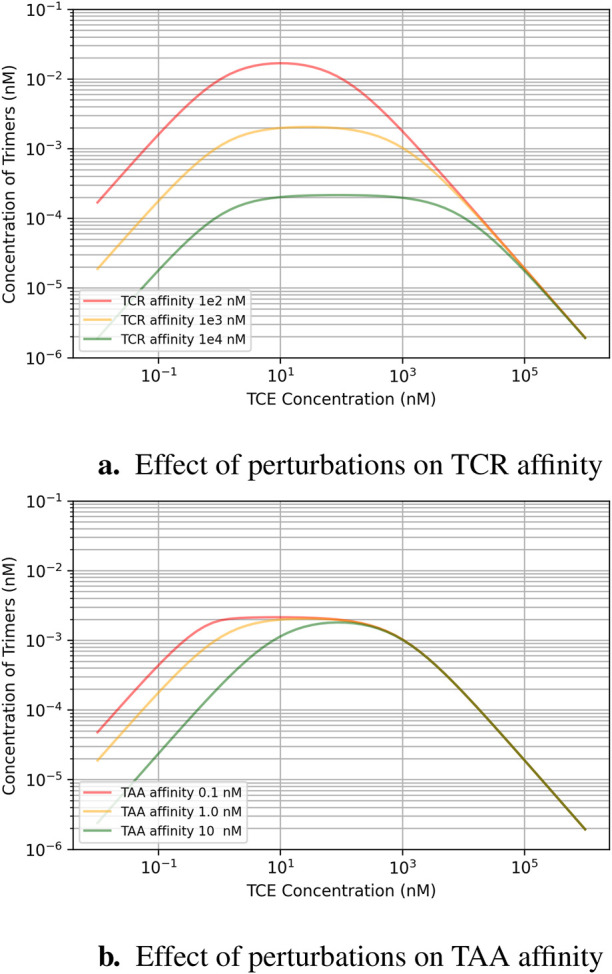
Effect of binding affinity perturbations around the ballpark design parameters recommended for TCEs (micromolar affinity for the TCR, robustly expressed at $10^{5}$ copies/cell, and nanomolar affinity for a TAA expressed at about $10^{4}$ copies/cell): **(A)** higher affinities for the TCR shift the dose-response curve both upwards and to the left; **(B)** higher affinities for TAA shift the dose response mainly to the left, extending the width of the response plateau.

This analysis aims to explain how factors like expression levels, the TCE binding affinity to its targets, and TCE exposure are interrelated, in particular when they are perturbed around the “classical” recommended values for TCE design. Figure 2A shows state equilibrium concentrations of dimers and trimers at varying concentrations of TCE. Figure 2B shows the relationship between predicted trimer formation and TCE concentration, given three different TAA expression level conditions. This effect may also be relevant to quantify loss of efficacy in the event of target downregulation after repeated dosing. The effect of a change of TCR affinity (*Kd* around the “typical” value of 1μM ) is illustrated in Figure 3A, while the effect of a change of affinity for TAA (*Kd* around the “typical” value of 1nM ) is illustrated in Figure 3B. Given that the affinity for the TAA is already high, a further increase would not boost peak trimer formation, but rather lower the exposure at which saturation is reached, whereas increasing TCR affinity could rescue low efficacy by having a more dramatic effect on peak trimer formation. As previously mentioned, however, this must be weighed against the increased risk of cytokine release, which poses a safety concern.

## 4 Discussion

A strong assumption underlying our modelling approach is that we are using TCR occupancy as a driver of efficacy. In naturally occurring immunological synapses, TCR occupancy is not sufficient for T cell activation. While TCR occupancy remains a necessary first step, co-stimulatory signals are also required. However, the synapse formed by TCEs is rather different from a physiological one. Usually the TCE targets directly a co-receptor subunit of the TCR complex, rather than the TCR central region itself. The interaction with the antigen is delegated to the second arm of the bispecific TCE, which binds directly to a surface antigen rather than an antigen peptide bound to an MHC. A salient feature of the artificial ternary synapses created by TCEs is their ability to induce cytotoxic responses independently from both MHC binding and co-stimulation (Bargou et al., 2008; Burt et al., 2019). To our knowledge, the resulting intracellular signaling is not well-mapped or quantitatively understood, but we believe it is fair to assume it is likely to differ from “standard” T-cell activation. In addition, since both *in vivo* and *in vitro* data show a clear exposure-response relationship, and since occupancy is clearly related to exposure, we decided to use occupancy as a crude but convenient proxy for what we may call “T-cell activation propensity”. Moreover, trimeric synapses have an important additional non-signalling function, namely, forcing cytotoxic T-cells to co-localize with their target cells.

A second simplifying assumption is the use of a well-mixed compartment to represent binding events. In fact, the second binding step happens between one free and one TCE-occupied receptor, floating on the membranes of two contiguous cells separated by a small gap of interstitial fluid. As pointed out by other authors, from a kinetic point of view, the net impact of membrane localization is not an obvious one, because although 2D diffusive searches are known to be more efficient than their 3D counterparts, which speeds up the reaction rate, diffusion in lipid bilayers restricts access to reactants and is orders of magnitude slower than in bulk solution, which slows down the reaction rate. Given the complexity of the problem and the current lack of specific data, we preferred to adopt the simplest possible approach, and defer a more thorough analysis to future work, possibly with agent-based models rather then ODE-based ones, as described for example by (Andrews, 2020).

Within the limitations outlined above, our results highlight a potentially critical factor involved in the design of effective TCE drugs. The optimal TCE exposure for creating the most effective trimer complexes (where the drug bridges the T-cell and cancer cell) relies mainly on the binding affinity between the TCE and the two targets: the expressed TAA on cancer cells and the T-cell receptor. Interestingly, the cancer receptor expression level in a patient does not influence the optimal TCE exposure: but it does affect the overall amount of trimer formation, it does not shift the exposure that yields the maximum trimer formation. A descriptive way of phrasing the effect of TAA expression levels is that they shift the dose-response curve “up and down” along the *y*-axis, but not “left and right” along the *x*-axis.

Since patient factors like T-cell concentration and TAA expression levels are not controllable, designing drugs with optimal target binding affinity becomes critical for clinical success. The safety requirement to minimize cytokine release risk by choosing TCEs with relatively low affinity for TCR creates an inevitable safety/efficacy trade-off that is best negotiated with the support of predictive models. This consideration should influence decisions about compound design and dosing strategies. On the other hand, selecting the right targets is essential as well. The TAA expression level directly affects the TCE exposure needed to activate enough T-cells for a potent immune response. If the TCE exposure is too low, there won’t be enough trimer formation to trigger a strong immune response. However, if the exposure is pushed beyond the ideal point, T-cell activation won’t necessarily increase due to more inactive dimer formation instead of active trimer formation–i.e., the system becomes saturated with dimers.

It is worth stressing that large molecules such as TCEs have more complex biodistribution patterns than small molecules (Xenaki et al., 2017), and local tissue exposure is most likely one of the main drivers of the target engagement mechanism that we discussed in this work. Therefore, a thorough understanding of how the drug distributes throughout the body and interacts with its targets on both cancer cells and T-cells should be prioritized from the very beginning of the design process. This knowledge will be instrumental in selecting the right binding strengths, choosing optimal targets, and ultimately, determining the most effective dosing strategies for TCE therapies.

## Data Availability

The original contributions presented in the study are included in the article/Supplementary Material, further inquiries can be directed to the corresponding author.
