# Supplementary material for: T-cell engagers: model interrogation as a tool to quantify the interplay of relative affinity and target expression on trimer formation
Source: Front Pharmacol. 2024 Oct 8;15:1470595. doi: 10.3389/fphar.2024.1470595 (PMC11493665; doi:10.3389/fphar.2024.1470595)
Supplement: Supplementary file 1 [file DataSheet1.PDF]

Supplementary Material

1 SUPPLEMENTARY TABLES AND FIGURES

1.1 Analysis of binding kinetics

Antibodies are known to have slow on-off kinetics, which can result in long equilibration times. To explore the validity of our steady state analysis, in particular the assumed k_{on} value of $10^6 M^{-1}s^{-1}$, we simulated the kinetics of equilibration up to a k_{on} two orders of magnitude below the hypothesized value. The results are summarised in Supplementary Figure S1. Furthermore, we tested how much a varying plasma PK profile would affect our steady-state predictions. Supplementary Figure S2 shows the predicted time profile for 24h post-dose, for a TCE with a 1 week half-life. Over a whole day, the concentration post-infusion would drop by only about 10%.

1.2 Perturbation analysis of the trimer formation curve

To assess the stability of the model with respect to parameter variations, the model was simulated 1000 times with randomly perturbed parameters. Each parameters was allowed to change up to two-fold. Only ”primary” (i.e. non-calculated) parameters were included in the perturbation analysis. These are summarised in Supplementary Table S1. The resulting shift in response curves is displayed in Supplementary Figure S3.

1.3 Tables

Table S1. Summary of affinity and other kinetic parameters used in the perturbation analysis. Sources are given in the main text

Parameter	Value	Units	Description
K_d^{TCR}	10^3	nM	TCE affinity for TCR
K_d^{TAA}	1	nM	TCE affinity for TAA
k_{on}^{TCR}	1e5	$M^{-1}s^{-1}$	Assoc. rate const. to TCR
k_{on}^{TAA}	1e5	$M^{-1}s^{-1}$	Assoc. rate const. to TAA
k_{deg}^{TCR}	1.834e-5	s^{-1}	Degradation rate of TCR
k_{deg}^{TAA}	1.834e-5	s^{-1}	Degradation rate of TAA
k_{int}^{TCR}	5.501e-5	s^{-1}	Internal. rate of bound TCR
k_{int}^{TAA}	5.501e-5	s^{-1}	Internal. rate of bound TAA
k_{int}^{Trm}	1.834e-5	s^{-1}	Degr. rate of trimer

1.4 Figures

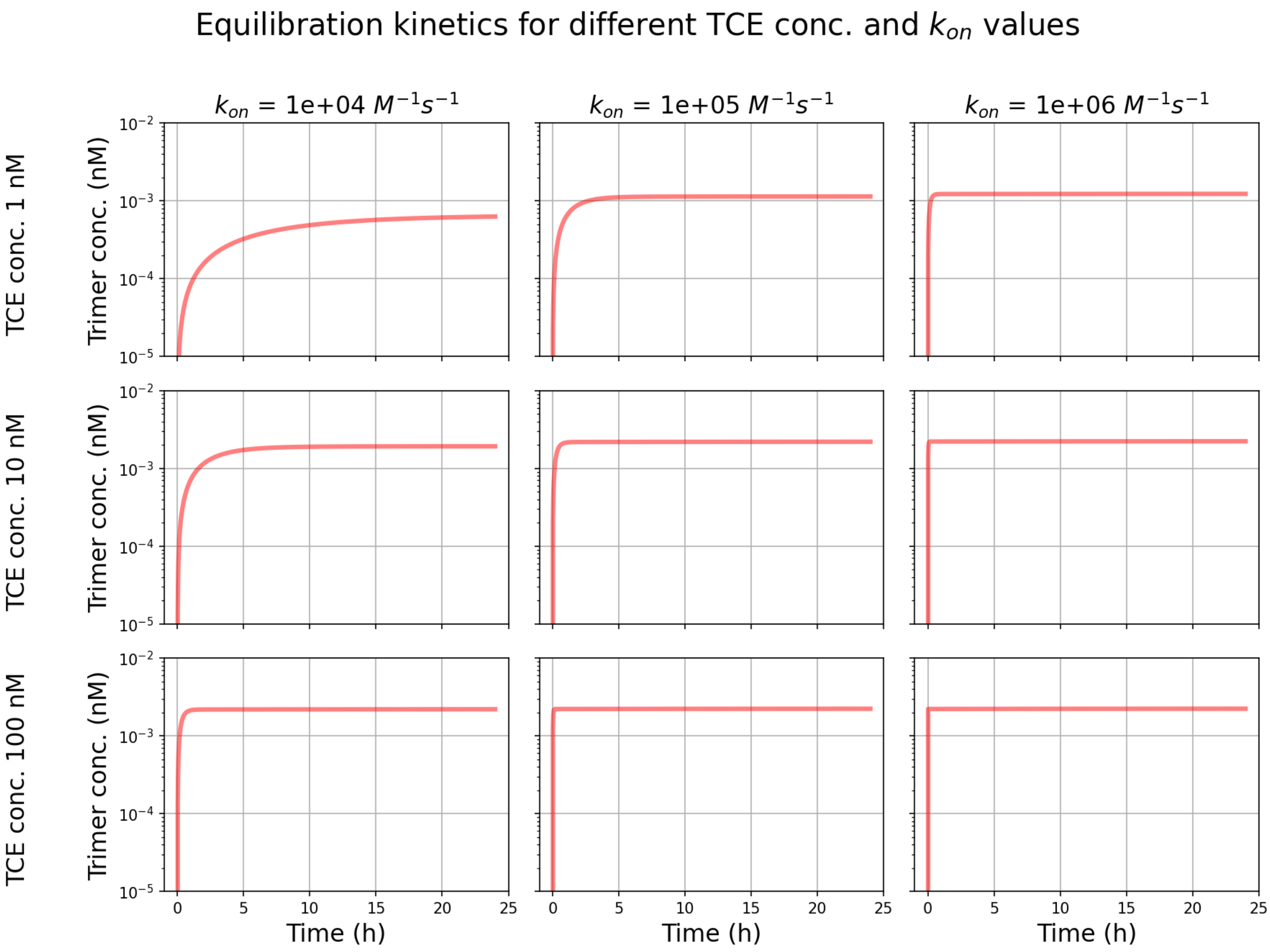

Figure S1. Simulation of equilibration kinetics for a range of k_{on} values between 1e4 and 1e6 $M^{-1}s_{-1}$ and a range of TCE concentrations from 1 to 100nM. The simulation results justify our assumption that in most conditions a steady state is reached within few hours.

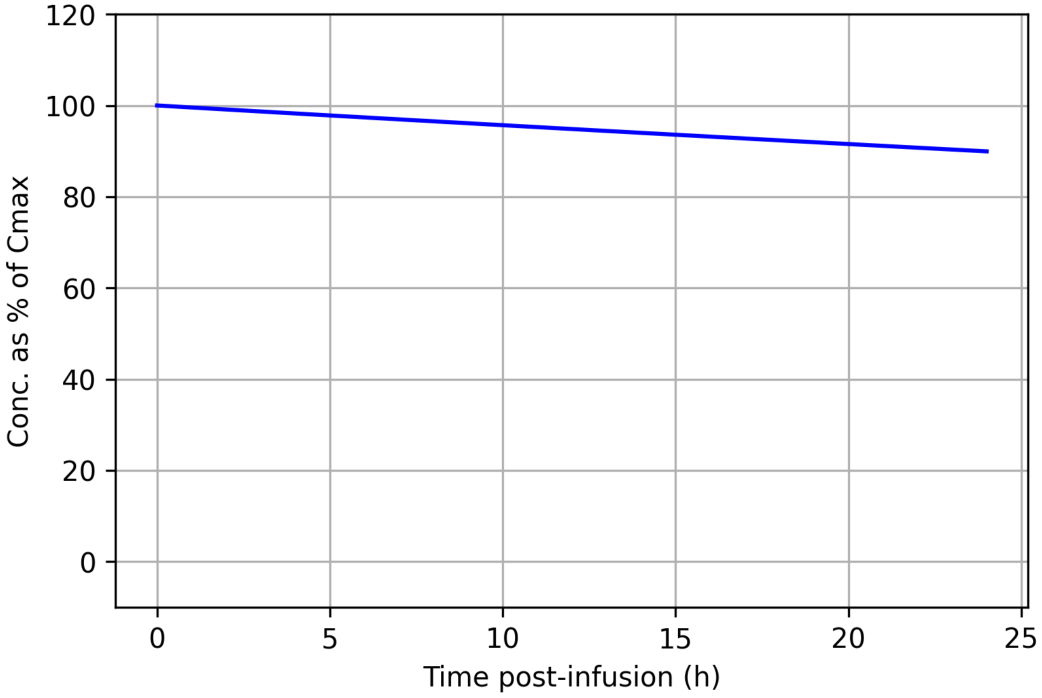

Figure S2. Time course of plasma concentration for 24h post-infusion, assuming linear clearance and a half-life of 7 days. the concentration on the y axis is expressed as % of Cmax, assuming that Cmax is reached early post-infusion.

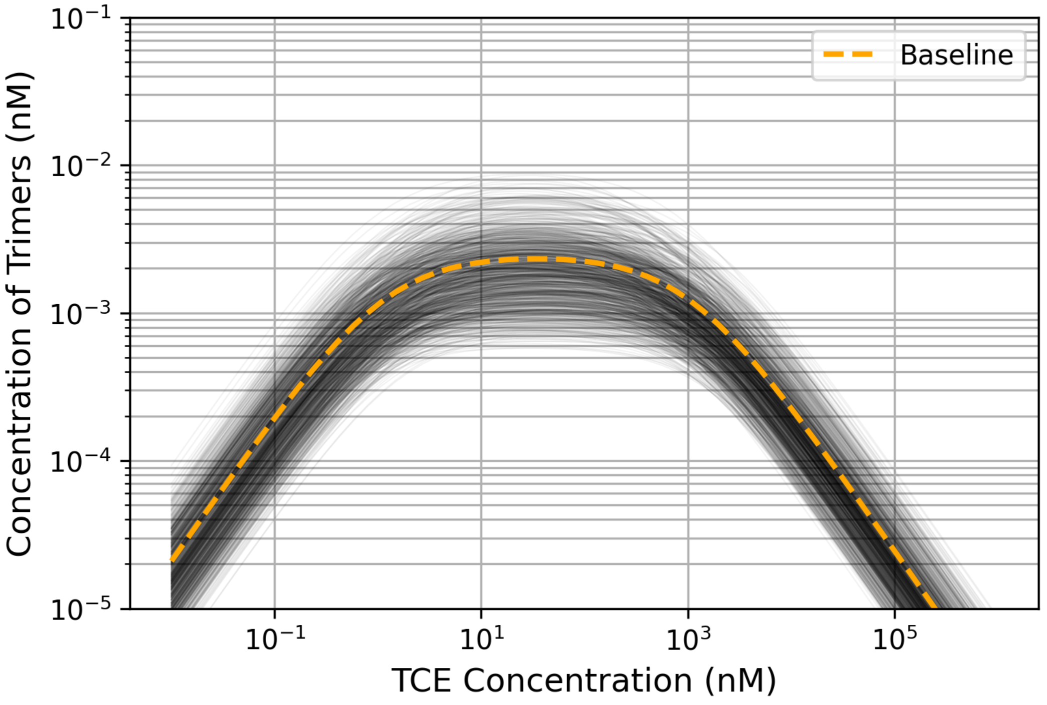

Figure S3. Plot of 1000 simulations with randomly perturbed model parameters (black lines) and comparison with baseline parameter values (orange dashes). Each parameter listed in Tab. S1 was allowed to vary up to two-fold.
